# Boosted Membrane Potential as Bioenergetic Response to Anoxia in *Dinoroseobacter shibae*

**DOI:** 10.3389/fmicb.2017.00695

**Published:** 2017-04-20

**Authors:** Christian Kirchhoff, Heribert Cypionka

**Affiliations:** Institute for Chemistry and Biology of the Marine Environment, Carl-von-Ossietzky University of OldenburgOldenburg, Germany

**Keywords:** proton-motive force, *Roseobacter* clade, Micrococcus luteus, short-term anoxia, intracellular pH, fluorescence microscopy, DiOC_2_(3), JC-10

## Abstract

*Dinoroseobacter shibae* DFL 12^T^ is a metabolically versatile member of the world-wide abundant Roseobacter clade. As an epibiont of dinoflagellates *D. shibae* is subjected to rigorous changes in oxygen availability. It has been shown that it loses up to 90% of its intracellular ATP when exposed to anoxic conditions. Yet, *D. shibae* regenerates its ATP level quickly when oxygen becomes available again. In the present study we focused on the bioenergetic aspects of the quick recovery and hypothesized that the proton-motive force decreases during anoxia and gets restored upon re-aeration. Therefore, we analyzed ΔpH and the membrane potential (ΔΨ) during the oxic-anoxic transitions. To visualize changes of ΔΨ we used fluorescence microscopy and the carbocyanine dyes DiOC_2_ (3; 3,3′-Diethyloxacarbocyanine Iodide) and JC-10. In control experiments the ΔΨ-decreasing effects of the chemiosmotic inhibitors CCCP (carbonyl cyanide m-chlorophenyl hydrazone), TCS (3,3′,4′,5*-*tetrachlorosalicylanilide) and gramicidin were tested on *D. shibae* and Gram-negative and -positive control bacteria (*Escherichia coli* and *Micrococcus luteus*). We found that ΔpH is not affected by short-term anoxia and does not contribute to the quick ATP regeneration in *D. shibae.* By contrast, ΔΨ was increased during anoxia, which was astonishing since none of the control organisms behaved that way. Our study shows physiological and bioenergetical aspects comparing to previous studies on transcriptomic responses to the transition from aerobic to nitrate respiration in *D. shibae*. For the lifestyle as an epibiont of a dinoflagellate, the ability to stand phases of temporary oxygen depletion is beneficial. With a boosted ΔΨ, the cells are able to give their ATP regeneration a flying start, once oxygen is available again.

## Introduction

*Dinoroseobacter shibae* is a member of the metabolically versatile Roseobacter clade and can be found in marine and fresh water habitats world-wide (Kolber et al., 2001; Béjà et al., 2002). It belongs to the aerobic anoxygenically phototrophic bacteria. Accordingly, it was documented that light supports proton translocation and therefore contributes to the ATP regeneration in *D. shibae* (Holert et al., 2011). However, the cells are able to switch from an aerobic to anaerobic lifestyle (Piekarski et al., 2009). As epibionts of dinoflagellates (Biebl et al., 2005) that can shuttle between oxic and anoxic conditions (Wagner-Döbler et al., 2010), this versatility may be helpful.

The oxic-anoxic shift causes dramatic changes of the cell physiology. Laass et al. (2014) reported that *D. shibae* undergoes a metabolic crisis during the transition from aerobic to anaerobic growth with nitrate. Transcriptomic analysis showed that protein biosynthesis in *D. shibae* is reduced during this phase. The energy balance is disturbed and accumulation of TCA-cycle intermediates can be observed until the cells adapt to nitrate as electron acceptor (Laass et al., 2014). Comparably, it was found that the cells lose up to 90% of their ATP when incubated anoxically for 2 h. However, they show remarkably fast ATP regeneration upon re-aeration and exposure to light. Within only 40 s the cells recovered by producing up to 12 mM of intracellular ATP (Holert et al., 2011, see also our experiments in Supplement 1).

The mechanisms behind the fast recovery of the energy charge after anoxia are not clear yet. In the present study we addressed the bioenergetics of short-term anoxia and the quick ATP recovery in *D. shibae.* We hypothesized that the proton-motive force is involved, assuming that it would be low in de-energized cells and increase during recovery. Therefore we analyzed ΔpH and ΔΨ during oxic-anoxic transitions. Membrane-potential disrupting ionophores and some other bacteria were used for control experiments. Analysis of the intracellular pH was performed via butanol permeabilization after Scholes and Mitchell (1970). Two carbocyanine dyes were used to visualize the membrane potential by fluorescence microscopy. As hypothesized we found changes of the proton-motive force, but quite different from our expectations.

## Materials and Methods

### Bacterial Strains and Cultivation

*Dinoroseobacter shibae* DFL 12^T^ (Biebl et al., 2005) was grown in artificial seawater medium (SWM) with 10 mM succinate as sole electron and carbon source (Supplement 2 for all media). Cells were cultivated in a diurnal light/dark rhythm (12 h/12 h, 12 μmol photons m^-2^ s^-1^) in a shaker at 25°C and 125 rpm (Soora and Cypionka, 2013). Control organisms *Escherichia coli* (strain K12) and *Micrococcus luteus* (strain E10-2, Batzke et al., 2007) were grown on LB medium at 33°C.

### Determination of the Intracellular pH (pH_i_)

The determination of the intracellular pH was performed after Scholes and Mitchell (1970) via permeabilization of the membrane with 5% (vol/vol) butanol. Cells were harvested by centrifugation (150 ml culture, 10.000 × *g*, 10 min, 4°C, Beckman J2-HS), the supernatant was discarded and the pellet was resuspended in 5 ml of non-buffered solution (0.3 M NaCl, pH 7). The measurement was performed in a 5 ml glass tube with an OD_436_ of 20. For the determination of the pH_i_ under anoxic conditions the glass tube was closed with a rubber stopper and flushed with N_2_ for 2 h. A pH electrode (type Inlab Micro, Mettler Toledo) was immersed and the suspension was stirred. The data were logged (AD converter ADC-16, pico Technology) with the software MPwin (version 2008.08.25, Cypionka, 2005). For permeabilization 250 μl of butanol were added into the stirred suspension. From the pH changes after butanol addition at different outer pH values, the pH_i_ could be determined.

### Application of Short-term Anoxia and Subsequent Re-aeration

Exponentially growing cells (5 ml) were taken directly for ΔΨ analysis. Additionally, two more aliquots (each 5 ml) were transferred into glass tubes closed by rubber stoppers. The tubes were permanently protected from light by aluminum foil during further treatment. Both tubes were flushed with N_2_ to quickly establish anoxic conditions. After 2 h incubation at 25°C, one of the tubes was flushed for 2 min with air under light exposure (420 μmol photons m^-2^ s^-1^) to allow recovery of the cells. The other tube was kept anoxic and dark. Afterward cells from both tubes were analyzed for their ΔΨ and compared to the untreated cells from before.

### Analysis of the Membrane Potential

The membrane potential was assessed via fluorescence microscopy with two ΔΨ-sensitive dyes, 3,3′-Diethyloxacarbocyanine Iodide [DiOC_2_(3), TCI, Germany] and JC-10 (AAT Bioquest, USA) using the same procedure. These lipophilic dyes form self-aggregates upon membrane uptake, which results in a color shift from green to red fluorescence. The appropriate concentrations of the dyes were figured out in a separate set of experiments, and were found to be 150 μM for *D. shibae* and *E. coli*, and 50 μM for *M. luteus*.

The staining procedure was performed with cells from three different energetic states; untreated cells from the exponential growth phase, cells that had been kept anoxic and dark for 2 h, and cells that had been re-aerated and exposed to light after anoxic and dark incubation.

During the following staining process, all cells were also protected from light to prevent photobleaching of the fluorescent dyes and unwanted, light-induced recovery of the cells. Therefore, 500 μl of the respective cells (OD_436_ 2.0) were transferred to 2 ml reaction tubes each, mixed with 500 μl PBS buffer and stained with DiOC_2_(3) for 15 min. For the untreated cells complete depletion of oxygen by respiration during staining could be excluded, even for maximum respiration rates (Holert et al., 2011). For the anoxically incubated cells anoxic conditions were maintained during staining by using N_2_-flushed PBS buffer and dye.

As a result of their double-layered cell membrane Gram-negative organisms like *D. shibae* tend to take up cyanine dyes worse than Gram-positives (Novo et al., 1999). To overcome this 1 mM EDTA was added prior to staining, which significantly improved the staining results for the Gram-negative strains *D. shibae* and *E. coli* (Dumas et al., 2010) by weakening the outer bacterial membrane (Alakomi et al., 2006). The Gram-positive strain *M. luteus* was used for control experiments.

After staining, 10 μl of the cell suspension was transferred onto object microscope slides and analyzed under a fluorescence microscope (Leica DMRB, Leitz) at excitation wavelength of 450–490 nm, dichroic filter 510 nm, longpass filter 515 nm (Leica-Nr.: 513808). At this excitation the normally green fluorescence (488–530 nm) shifts to red (>600 nm) when the dye is taken up by the cells due to their membrane potential (Novo et al., 1999). Pictures were taken with an EOS 600 D camera using the EOS utility software (ver. 2.10.2.0). Final image processing was performed with PICOLAY (Cypionka et al., 2016). The quantification of the % of red stained cells was done with CountThem (Cypionka, 2011).

### ΔΨ-Dissipating Agents

Carbonyl cyanide m-chlorophenyl hydrazone (CCCP), 3,3′,4′,5*-*tetrachlorosalicylanilide (TCS) and gramicidin were used for the disruption of ΔΨ. Samples were treated as described before and subsequently analyzed with fluorescent microscopy. The ionophores were added prior to staining and the samples were incubated 5 min at room temperature in the dark. Applied concentrations were 2 μM CCCP, 2 μM TCS, and 5 μg/ml gramicidin. In control experiments, the solvent DMSO did not have any influence without the inhibitors.

## Results

### The Intracellular pH of *Dinoroseobacter shibae* Is Independent of the Energetic State

**FIGURE 1:**
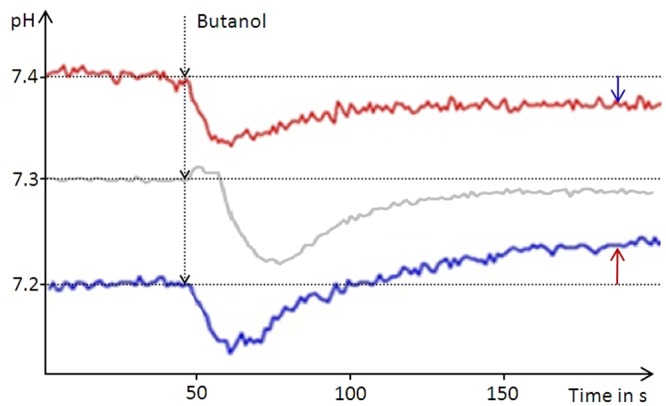
**Narrowing down the intracellular pH of *D. shibae.*** The pH of an unbuffered cell suspension was constantly recorded during butanol treatment leading to leakage of the cells. Arrows indicate the direction of the pH change from the respective starting pH of the suspension (red graph 7.4, blue graph 7.2) toward the intracellular pH of 7.3. At the starting pH of 7.3 the pH change is very small and can vary between the experiments.

In order to determine the intracellular pH (pH_i_) of *D. shibae* we used butanol (5% vol/vol) for membrane permeabilization. Butanol treatment resulted in alkalinization of the suspension when the initial pH was below 7.2 and in acidification of the suspension at pH values above 7.4. For the initial pH of 7.3 only minimal changes were observed after butanol treatment, varying slightly between repeated experiments (**Figure 1**). Thus, the pH_i_ was determined to lie between 7.2 and 7.4. This result was identical for all *D. shibae* cells, including untreated-, anoxically incubated- and subsequently re-aerated cells. This indicates that the pH_i_ in *D. shibae* is independent of the energetic state. For *E. coli* a pH_i_ between 7.5 and 7.6 was determined with the same technique, confirming literature values (Wilks and Slonczewski, 2007).

### Short-term Anoxia Causes a Boost of the Membrane Potential of *Dinoroseobacter shibae*

**FIGURE 2:**
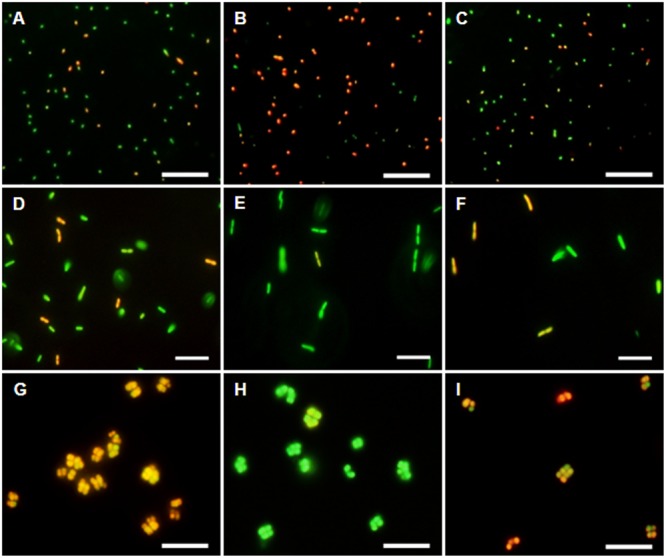
**DiOC_2_(3)-uptake at different energetic states.** (Left column) Untreated cells in exponential growth phase. (Middle column) Cells after 2 h of N_2_-gassing. (Right column) Cells after 2 h N_2_-gassing, aerated for 2 min subsequently. **(A–C)** Dye uptake of *D. shibae* increases after N_2_-gassing but declines after subsequent aeration. **(D–F)** Dye uptake of *E. coli* decreases after N_2_-gassing, but increases after subsequent aeration. **(G–I)** Dye uptake of *M. luteus* was strongest at the start, decreased drastically after N_2_-gassing, but increases after subsequent aeration. All scales: 10 μm.

**FIGURE 3:**
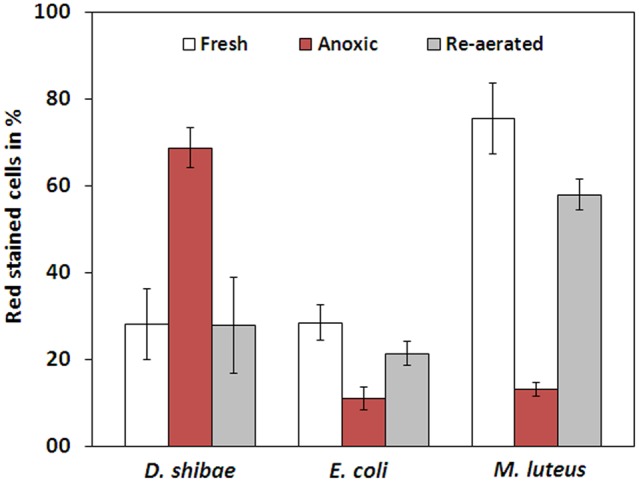
**Fraction of red stained cells for fresh, anoxic, and re-aerated cells.** *D. shibae* has 2.5-fold more red stained cells during anoxia, compared to fresh cells and after re-aeration. *E. coli* has 2.5-fold and *M. luteus* even 5-fold less red stained cells during anoxia. All strains approach their original value after re-aeration.

The membrane potential was visualized with two fluorescent carbocyanine dyes, DiOC_2_(3) and JC-10. The accumulation of DiOC_2_(3) underwent major changes between oxygen availability and anoxia. Freshly harvested cells of *D. shibae* stained moderately red, indicating the standard state of the membrane potential (**Figure 2A**). After anoxic incubation, cells stained more intensely red indicating increased ΔΨ (**Figure 2B**). Cells that were aerated after anoxic incubation, again showed moderate dye uptake and red fluorescence, similar to untreated cells (**Figure 2C**). A quantitative measure by image analysis shows that the amount of red stained cells increases about 2.5-fold during anoxia (**Figure 3**). These results were confirmed with the alternative fluorescent dye JC-10, although the fluorescence was a little weaker in intensity as with DiOC_2_(3) (Supplement 3). This shows that the uptake and intracellular accumulation of ΔΨ-dependent dyes in *D. shibae* changes significantly between oxic and anoxic conditions suggesting an increased ΔΨ in anoxic cells. The outcome of the staining was independent of the presents or absence of succinate, which was used as the cells sole electron and carbon source during cultivation. Furthermore, the increase of ΔΨ during anoxia could be observed for varying external pH values. With a pH of 7.5, the growth medium of *D. shibae* is more alkaline than the pH_i_ (near 7.3). We obtained the same results with cells that were grown and tested at pH 7.0. Even at an external pH of 6.5, which is slightly below the pH optimum of *D. shibae* (Biebl et al., 2005), the effect was still observed. However, these cells appeared more orange than red. (Supplement 4).

### Bacteria in Control Experiments Showed Stable- or Decreased Δ**Ψ** during Anoxia

The observation of an increasing ΔΨ for *D. shibae* under anoxic conditions was unexpected. To clarify this behavior we analyzed the ΔΨ of other bacteria under the same conditions. *E. coli* revealed a decrease in ΔΨ during short-term anoxia (**Figures 2D–F**) with about 2.5 times less red stained cells (**Figure 3**). The strictly aerobe *M. luteus* (Woodward and Kell, 1991), which took up the dye very well from the start, showed a drastically decreased ΔΨ after N_2_-gassing (**Figures 2G–I**), with five times less red stained cells (**Figure 3**). Unlike *D. shibae*, none of the tested control organisms showed an increased ΔΨ during oxic or anoxic conditions.

### Protonophore Treatment Proves Δ**Ψ**-Depending Dye Uptake

**FIGURE 4:**
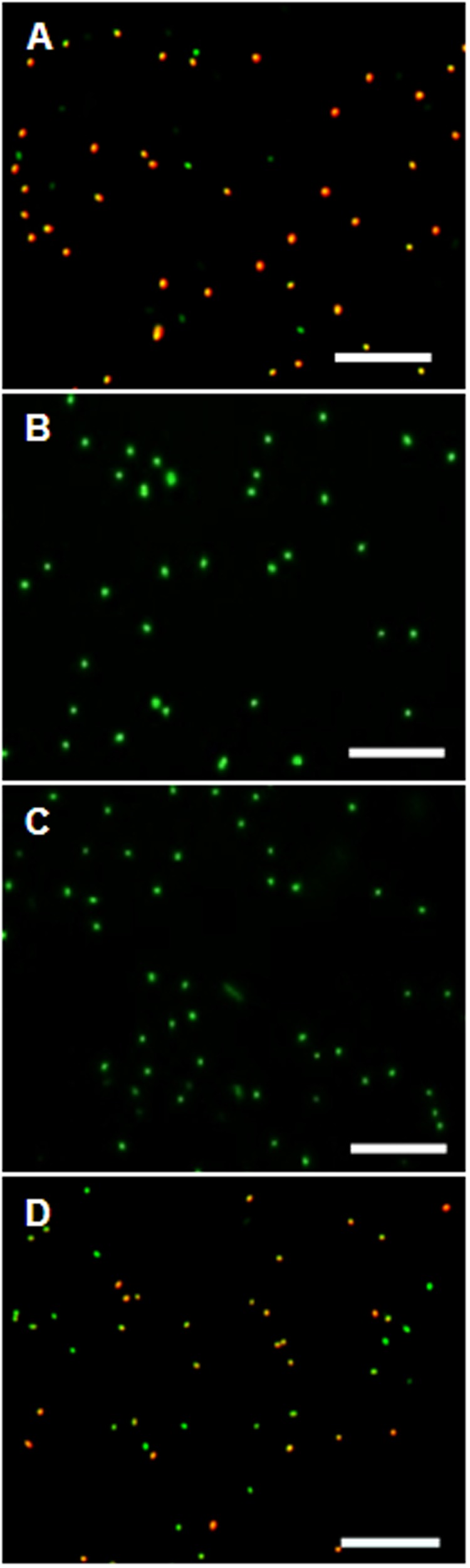
**The effect of Δ**Ψ**-dissipating agents on the DiOC_2_(3)-uptake of *D. shibae* cells. (A)** *D. shibae* cells with increased membrane potential. **(B)** 2 μM CCCP. **(C)** 5 μM TCS. **(D)** 5 μg/ml gramicidin. All scales: 10 μm.

Control experiments with protonophores were conducted to make sure the observed uptake of the dye is indeed ΔΨ-depending. In all tested organisms dye uptake was drastically decreased and red fluorescence was prevented almost completely when the cells were pre-incubated with the protonophores CCCP or TCS for 5 min (**Figure 4**). The channel-forming peptide gramicidin did not work as effective for the Gram-negative bacteria *D. shibae* and *E. coli*, but worked fine on the Gram-positive *M. luteus*. With gramicidin, the amount of *D. shibae* cells stained red/orange decreased from previously mentioned 69(±5)% to 41(±1)%. For *M. luteus* all cells appeared green with no visible dye aggregation.

## Discussion

In the present study, we show that the membrane potential ΔΨ of *D. shibae* is strongly influenced by short-term anoxia – although in an unexpected way – while ΔpH is not affected. The used carbocyanine dyes for ΔΨ-visualization are usually applied in combination with flow cytometry (Novo et al., 1999; Jiao et al., 2004; Zhen et al., 2014). We have shown that fluorescence microscopy can be a useful alternative, since it produces *in vivo* pictures of ΔΨ-caused dye-uptake (Supplement 5).

### ΔpH Is Not Contributing to ATP Recovery

Different from our expectations, ΔpH is seemingly not contributing to the quick ATP recovery of *D. shibae.* Instead the intracellular pH was determined to be near 7.3, and was not affected from short-term anoxia and re-aeration. The intracellular pH was lower than that of the medium (pH 7.5), resulting even in a slightly reversed ΔpH. We conclude that ΔpH does not support proton-driven ATP synthesis in *D. shibae.* Instead, our results suggest that the proton-motive force relies mainly on ΔΨ.

### Boosted Membrane Potential Supports ATP Regeneration

According to our expectation short-term anoxia had an impact on ΔΨ. However, *D. shibae* increased its membrane potential as reaction to short-term anoxia and the resulting ATP depletion, while ΔpH remained unchanged. An increasing membrane potential as a response to anoxia is unusual, as indicated by the analyzed control organisms. None of them showed a comparable reaction to short-term anoxia. It was shown before that *D. shibae* cells undergo a metabolic crisis during the absence of oxygen and metabolites start to accumulate (Laass et al., 2014). We show that this crisis is also reflected on a bioenergetic and physiological level. The previously documented drop in the ATP content (Holert et al., 2011) and the newly found increase in membrane potential are tied together and a result of the cells’ adaptation to anoxia. Like the metabolic crisis, the increased ΔΨ normalizes with re-aeration. However, it is still unclear how the process of increasing ΔΨ works in detail. Our results suggest an electrogenic mechanism as a response to anoxia. Apparently, the process is not electron-donor dependent, as it was independent of succinate availability.

A boosted membrane potential can give the ATP regeneration of *D. shibae* a flying start, once oxygen becomes available again. The membrane potential is known to be the more potent component of the proton-motive force for bacteria that live in pH-neutral environments (Krulwich et al., 2011). The bioenergetic kickstart is beneficial for *D. shibae* with respect to its natural environment. Diurnal alterations in oxygen availability are very common in phototrophic biofilms (Steunou et al., 2008). *D. shibae* is abundant in this habitat as a dinoflagellate epibiont (Biebl et al., 2005; Parsons and Preskitt, 2007) and being able to adapt to these alteration is advantageous for its survival (Wagner-Döbler et al., 2010).

However, ΔΨ cannot be solely responsible for the observed ATP regeneration capacity. It is build up by a remarkably low amount of charges per cell and has about -135 mV in its normal state (Krulwich et al., 2011). Assuming the cell size described by Biebl et al. (2005) and the standard membrane capacity (1 μF/cm^2^, Adrian, 1980), about 33 10^3^ charges would be required to increase ΔΨ by -180 mV. With an H^+^/ATP ratio of 4 (Elston et al., 1997) the cell could regenerate about 48 μM of intracellular ATP. In order to regenerate 12 mM of ATP (Holert et al., 2011), the process must be quickly supported by respiration- or light-driven proton translocation.

The increased ΔΨ during anoxia cannot possibly be caused by the ΔpH. A ΔpH value of 0.5 could at maximum builds up 30 mV membrane potential. We observed the ΔΨ increase at favorable and unfavorable ΔpH values above and below the intracellular pH. This demonstrates that the documented effect is significantly larger than 30 mV and based on a different mechanism.

## Author Contributions

CK concept, experiments, data analysis, and wrote manuscript. HC concept and wrote manuscript.

## Conflict of Interest Statement

The authors declare that the research was conducted in the absence of any commercial or financial relationships that could be construed as a potential conflict of interest.
