# Supplementary material for: Boosted Membrane Potential as Bioenergetic Response to Anoxia in Dinoroseobacter shibae
Source: Front Microbiol. 2017 Apr 20;8:695. doi: 10.3389/fmicb.2017.00695 (PMC5397407; doi:10.3389/fmicb.2017.00695)
Supplement: Supplementary file 1 [file Data_Sheet_1.DOCX]

**Supplementary material**

**Boosted membrane potential as bioenergetic response to anoxia in *Dinoroseobacter shibae***

Christian Kirchhoff^1^ and Heribert Cypionka^1^

^1^Institute for Chemistry and Biology of the Marine Environment (ICBM), Carl-von-Ossietzky University of Oldenburg, Oldenburg, Germany

**Supplement 1 – ATP extraction and determination**


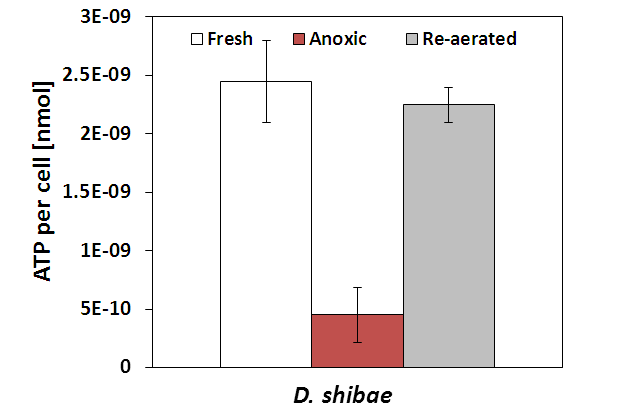


**ATP concentration for fresh-, anoxic and re-aerated *D. shibae* cells.** Fresh cells contained about 2.5 nmol ATP per cell (9.5 mM). After anoxic incubation this value dropped drastically to 0.5 nmol ATP (1.4 mM). Subsequent re-aeration and light exposure allowed the cells to regenerate to 2.4 nmol ATP per cell (8.8 mM).

The ATP was extracted with PCA according to the protocol of Blaut and Gottschalk (1984). After the different treatments of aliquot fresh, anoxic and re-aerated, 200 μl of each suspension were diverted and put into 200 μl ice cold PCA. After 1.5 hours of cold extraction, 200 μl of 3 M KOH and 100 μl 1 M TES-buffer were added. The precipitating perchlorate was centrifuged with a tabletop centrifuge and the supernatant was transferred to a new Eppendorf cup. The pH was determined with pH indicator paper (Merck) and adjusted to 7.0 – 8.0 with KOH and TES. The exact volume of each adjustment step was noted to calculate the correct dilution for every sample.

With a luciferin-luciferase reaction and the detection of the emitted light, the ATP concentration of the samples was determined, according to the protocol of Bergmeyer (Gruber W., Moellering H. and Bergmeyer HU., 1966). Therefore, the samples were analyzed within a luminometer (LKB, Wallac), and data were recorded by MPwin (vers. 2008.08.25, Cypionka).

The luciferase solution and the ATP standard were taken from the ATP Bioluminescence Assay Kit CLS II (Roche). 160 μl of 0.1 M TES-buffer and 40 μl of luciferase solution were applied to a cuvette. This was taken as the blank value. 2.5 μl of the sample were added. After reaching a stable signal, 2.5 μl of ATP standard was added, which was repeated once, after the signal was stable again. The ATP concentration of a single cell was determined, using a prolate spheroid cell model (Hillebrand *et al.* 1999) with a diameter of 1 μm.

Literature

Blaut, M. and Gottschalk, G. (1984) Coupling of ATP synthesis and methane formation from methanol and molecular hydrogen in *Methanosarcina barkeri*. *European Journal of Biochemistry* 141, 217-222

Cypionka, H. (2005). Mpwin software. Vers. 2008.08.25. www.pmbio.icbm.de/downlist.htm

Gruber, W., Moellering, H., Bergmeyer, H.U. (1966). Analytical differentiation of purine and pyrimidine nucleotides. I. Determination of AMP, ADP and ATP, and of GTP + ITP. *Enzymologia biologica et clinica (Basel),* 7(1):115-29

Hillebrand, H., Dürselen, CD., Kirschtel, D., Pollingher, U., Zohary, T. (1999) Biovolume calculation for pelagic and benthic microalgae. *Journal of Phycology,* 35, 403–424

**Supplement 2 – Growth media**

Artifical seawater medium (SWM):

SWM-basis (5x):

NaCl 100 g

Na_2_SO_4_ 20 g

MgCl_2_ · 6 H_2_O 15 g

KCl 2.5 g

NH_4_Cl 1.25 g

KH_2_PO_4_ 1 g

CaCl_2_ · 2H_2_O 0.75 g

Dissolve in 800 mL dH_2_O, then bring the volume to 1000 mL with dH_2_O and autoclave for 20 min at 121°C.

NaHCO_3_ stock solution (100x):

1.9 g NaHCO_3_

Dissolve in 100 ml dest. H_2_O and autoclave for 20 min at 121°C.

0.5 M Succinate solution (100x):

Dissolve 29.52 g succinic acid (M = 118.09 g/mol) in 400 ml dH_2_O. Adjust pH value with NaOH to pH 7.5. Bring to the volume of 500 ml with dH_2_O and autoclave for 20 min at 121°C.

| Trace element solution (1000 ml, 1000x): | |  |
| --- | --- | --- |
| H_2_O | 50 ml | |
| FeSO_4_ · 7H_2_O | 2.1 g | |
| 25% HCl | 13 ml | |
| Titriplex III (Na_2_EDTA**)** | 5.2 g | |
| H_3_BO_3_ | 30 mg | |
| MnCl_2_ · 4H_2_O | 100 mg | |
| CoCl_2_ · 6H_2_O | 190 mg | |
| NiCl_2_ · 6H_2_O | 24 mg | |
| CuCl_2_ · 2H_2_O | 2 mg | |
| ZnSO_4_ · 7H_2_O | 144 mg | |
| Na_2_MoO_4_ · 2H_2_O | 36 mg | |

Autoclave for 15 min at 121°C

Vitamin stock solution (100x):

Biotin 2 mg

Nicotinic acid 20 mg

4-aminobenoic acid 8 mg

Dissolve in 100 mL dest. H_2_O and filter-sterilize (0.2 µm pore size).

To obtain 100 ml SWM 10 mM succinate, compound the stock solutions as follows:

H_2_O, dest, autoclaved 73.9 ml

SWM-Basis 20 ml

Succinate stock 2 ml

NaHCO_3_ stock 3 ml

Vitamin stock solution 1 ml

Trace element solution 100 µl

Set the pH to 7.5 with HCl and KOH. The optimal growth range for *D. shibae* strain DFL 12^T^ is between 6.5 and 8.8 (Biebl *et al.* 2005).

Lysogeny broth (LB medium):

Bacto trypton 10.0 g

Bacto yeast extract 5.0 g

NaCl 10.0 g

Dissolve in 800 mL dH_2_O, set the pH to 7.0 with HCl and KOH, then bring the volume to 1000 ml. Autoclave for 20 min at 121°C. For agar plates add 12 g agar.

Literature

Biebl, H., Allgaier, M., Tindall, B. J., Koblizek, M., Lünsdorf, H., Pukall, R. and Wagner-Döbler, I. (2005). *Dinoroseobacter shibae* gen. nov., sp. nov., a new aerobic phototrophic bacterium isolated from dinoflagellates. *Int. J. Syst. Evol. Microbiol*. 55, 1089–1096. doi: 10.1099/ijs.0.63511-0

**Supplement 3 – JC-10-uptake at different energetic states**

**
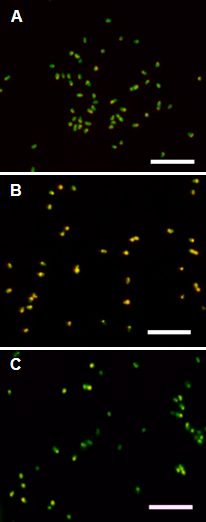
**

JC-10-uptake of *D. shibae* at different energetic states. (A) Fresh cells in exponential growth phase. (B) Cells after 2 h of N_2_-gassing. (C) Cells after 2 h N_2_-gassing, aerated for 2 min subsequently. All scales: 10 µm.

**Supplement 4 – Increased dye-uptake during anoxia at different external pH values**

**
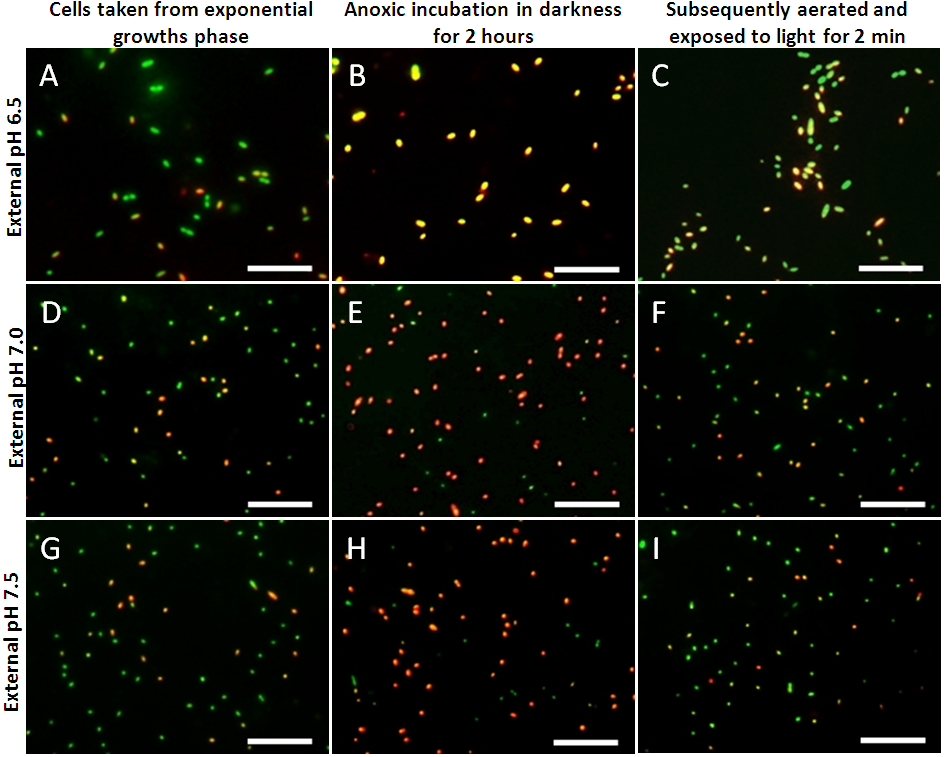
**

DiOC_2_(3)-uptake of *D.shibae* at different energetic states and external pH values. (Left column) Untreated cells in exponential growth phase. (Middle column) Cells after 2 h of N_2_-gassing. (Right column) Cells after 2 h N_2_-gassing, aerated for 2 min subsequently. (A-C) Dye uptake of *D. shibae* increases after N_2_-gassing but declines after subsequent aeration at an external pH of 6.5. The observed effect is weaker compared to external pH 7.0 and 7.5 (D-F) Dye uptake of *D. shibae* increases after N_2_-gassing but declines after subsequent aeration at an external pH of 7.0. (G-I) Dye uptake of *D. shibae* increases after N_2_-gassing but declines after subsequent aeration at an external pH of 7.5. All scales: 10 µm.

**Supplement 5 – DiOC_2_(3)-aggregation in *Bacillus subtilis***


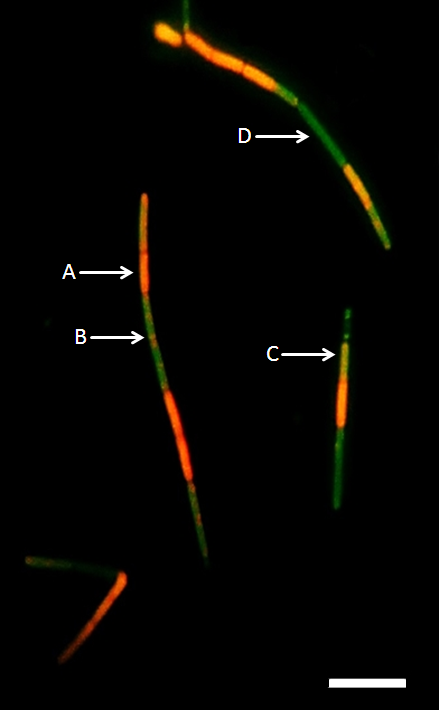


Exemplary DiOC_2_(3)-aggregation within cells of an early exponential growth phase *B. subtilis* culture. (A) Cell with highly polarized membrane, resulting in massive dye uptake and red staining. (B) Cell with weakly polarized membrane. Only small dye aggregates forming. (C) Cell with moderate dye accumulation, resulting in an orange/yellow staining. (D) Cell with nearly no dye uptake, no accumulation results in faint green staining. Scale 10 µM.
